# Evaluation of the Specific Activity of M−N−Cs and the Intrinsic Activity of Tetrapyrrolic FeN_4_ Sites for the Oxygen Reduction Reaction

**DOI:** 10.1002/anie.202207089

**Published:** 2022-11-10

**Authors:** Davide Menga, Ana Guilherme Buzanich, Friedrich Wagner, Tim‐Patrick Fellinger

**Affiliations:** ^1^ Bundesanstalt für Materialforschung und -prüfung (BAM) 12203 Berlin Germany; ^2^ Chair of Technical Electrochemistry Department of Chemistry and Catalysis Research Center Technische Universität München (TUM) 85748 Garching Germany; ^3^ Department of Physics Technische Universität München (TUM) 85748 Garching Germany

**Keywords:** M−N−C Catalysts, Oxygen Reduction Reaction, Tetrapyrrolic FeN_4_ Sites, Turnover Frequency, Utilization

## Abstract

M−N−C electrocatalysts are considered pivotal to replace expensive precious group metal‐based materials in electrocatalytic conversions. However, their development is hampered by the limited availability of methods for the evaluation of the intrinsic activity of different active sites, like pyrrolic FeN_4_ sites within Fe−N−Cs. Currently, new synthetic procedures based on active‐site imprinting followed by an ion exchange reaction, e.g. Zn‐to‐Fe, are producing single‐site M−N−Cs with outstanding activity. Based on the same replacement principle, we employed a conservative iron extraction to partially remove the Fe ions from the N_4_ cavities in Fe−N−Cs. Having catalysts with the same morphological properties and Fe ligation that differ solely in Fe content allows for the facile determination of the decrease in density of active sites and their turn‐over frequency. In this way, insight into the specific activity of M−N−Cs is obtained and for single‐site catalysts the intrinsic activity of the site is accessible. This new approach surpasses limitations of methods that rely on probe molecules and, together with those techniques, offers a novel tool to unfold the complexity of Fe−N−C catalyst and M−N−Cs in general.

## Graphical abstract

We herein used the equivalence of an active site extraction to the poisoning of active sites to develop a new method for the determination of site density, utilization factor and TOF. The method is reminiscent of stripping techniques but since there are no probe molecules involved it can be applied to different classes of M−N−C directly at the relevant pH.

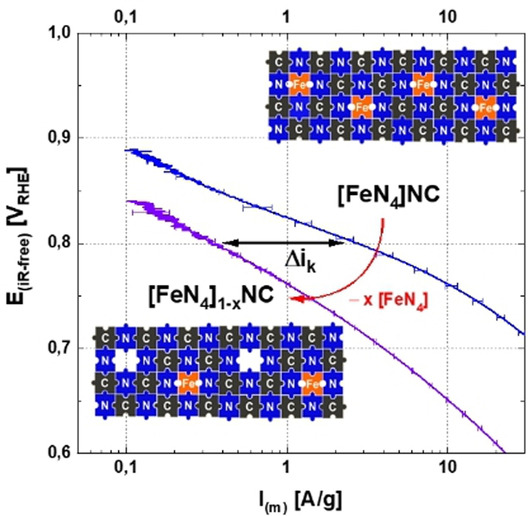

In the field of electrochemistry, metal and nitrogen co‐doped carbons (M−N−C) have attracted the interest of the community due to their application in many relevant energy conversion processes, such as oxygen reduction reaction (ORR),^[1]^ CO_2_ reduction reaction (CO_2_RR)^[2]^ and ammonia electrosynthesis.^[3]^ In order to replace expensive precious group metals (PGM) electrocatalysts, M−N−C need to meet their activity and stability, or at least compensate with their reduced cost. This seems to be the case especially for the ORR in proton exchange membrane fuel cells (PEMFCs) where Fe−N−C approaches the activity of expensive Pt‐based electrocatalyst^[4]^ and their stability improvement towards system‐relevant levels is becoming the focus of the community.^[5]^ The improved understanding of these materials is pushing their development forward and recently synthetic procedures have been optimized to produce exclusively the desired FeN_4_ active site free from less active inorganic side phases.^[6]^ In our previous work, we showed that via an active‐site imprinting strategy followed by a transmetalation reaction, Mg−N−C and Zn−N−C containing Mg−N_4_ and ZnN_4_ sites respectively, can be transformed into active Fe−N−C electrocatalysts, avoiding the formation of elemental iron, or iron carbide side phases.^[7]^ Moreover, when Zn is employed, the nature of the metal‐coordinating nitrogen atoms is pyrrolic and very active and highly selective tetrapyrrolic Fe−N−Cs can be prepared.^[6b]^ This newly developed synthetic method has proven able to surpass previous limitations in metal loading and to elucidate structure‐performance relations. In fact, the most active Fe−N−C electrocatalysts for ORR reported to date are made via the transmetalation of ZnN_4_ sites of a zeolitic imidazolate framework (ZIF)‐based Zn−N−C material into FeN_4_ sites.^[8]^

Since the effective activity of catalysts is overlayed with morphological features,^[9]^ rationally comparing the activity of morphologically different catalysts is complicated and not always meaningful. The quantification of specific activity like mass activity and turnover frequency (TOF) is therefore desirable. Due to the difficulties in assigning the nature of active phase/sites, for Fe−N−C these parameters have been elusive for many years. Since the community agrees on atomically dispersed FeN_4_ sites as most active phase, lately two methods have been proposed and are mainly employed, namely nitrite stripping, firstly reported by Kucernak et al.,^[10]^ and CO cryo‐sorption, introduced by Strasser et al.^[11]^ In order to overcome their limitation, these two methods have been used in a complementary way and this approach has proven fundamental in quantifying the SD and TOF of FeN_4_ active‐sites.^[12]^ Even more recently, an in situ electrochemical method based on Fourier‐transform alternating current voltammetry has been introduced.^[13]^ Herein, we report a facile and accessible method that surpasses current limitations in the determination of SD and TOF, which is also applicable to M−N−Cs in general, not only to Fe−N−Cs. Moreover, we employ the new method to a catalyst that can be approximated as single‐site catalyst revealing the intrinsic catalytic activity of tetrapyrrolic FeN_4_ sites.

The pristine catalyst presented in this study is prepared by carbonizing 1‐ethyl‐3‐methylimidazolium dicyanamide (Emim‐dca) in a ZnCl_2_/NaCl eutectic mixture (*T*_m_=250 °C) at 900 °C in Ar atmosphere (for details see Supporting Information). This salt templating strategy allows to achieve high yields and the final morphology of the catalyst can be finely tuned by simply adjusting the mixture composition.^[14]^ Moreover, the presence of the Lewis‐acidic Zn^2+^ ions facilitates the formation of N_4_ moieties.^[7b]^ The precursor to salt ratio was chosen based on previous work in order to obtain an aerogel‐like structure, which allows for high surface area and efficient mass‐transport.^[15]^ After low‐ and high‐temperature Zn‐to‐Fe ion‐exchange reaction,^[6b]^ the final catalyst is obtained. Two isomorphic partially Fe‐extracted catalysts are then obtained after partial extraction of Fe from the FeN_4_ sites of the pristine Fe−N−C catalyst using 2.4 M HCl, a non‐oxidizing acid, and moderate temperatures of 100 °C for different times. Based on their elemental composition, samples are named with their approximate stoichiometry with emphasis on the active‐ and Fe‐removed FeN_4_ site, i.e. [FeN_4_]N_32_C_520_ indicates the pristine sample and [FeN_4_]_*x*_[N_4_]_1−*x*_N_32_C_520_ the partially Fe‐extracted ones (*x*=0.23 and 0.17, respectively). The composition obtained from elemental combustion analysis is confirmed by scanning electron microscopy energy‐dispersive X‐ray spectroscopy (SEM/EDX) spectra (Figure S1) and small impurities of Al and Si, most likely due to the crucible and lid employed in the synthesis, are also found. The presence of Cl is attributed to the washing of the sample. Importantly, [FeN_4_]_*x*_[N_4_]_1−*x*_N_32_C_520_ samples do not present more Cl compared to the pristine sample, excluding functionalization of the carbon scaffold from the HCl treatment employed to remove the Fe. Moreover, the Fe content in the Fe‐extracted samples is too little to be detected in the SEM/EDX analysis. SEM images show the aerogel‐like structure of the catalysts before and after Fe extraction (Figure 1c). The small and connected particles create a hierarchical pore structure with a large surface area, which is confirmed by nitrogen‐sorption porosimetry measurements. As depicted in Figure 1a, [FeN_4_]N_32_C_520_ shows a large N_2_ uptake at low relative pressure, indicating the microporous structure of the primary particles. The mixed type II‐type IV isotherm is indication of the meso‐ and macroporosity of the material and its large interstitial porosity between the moderately aggregated particles,^[16]^ resulting in a very high apparent surface area of 2067 m^2^ g^−1^, perspective of very accessible active sites. A QSDFT model for slit and cylindrical pores was employed to calculate the pore size distribution from the adsorption branch of the isotherms (Figure 1b). Qualitatively, no substantial change in mesoscale pore size distribution is observed, pointing to the retained morphology of the material after Fe removal. Small deviations at around 1 nm point to local nanoscopic rearrangements connected to the decomplexation (Figure S2). However, no substantial morphological effect on the activity is expected to arise.^[9]^ The slightly lower N_2_ uptake at higher relative pressure and larger pore sizes is attributed to a reduction of the interstitial distance between the primary particles. This may be ascribed to partial collapse of the aerogel structure towards a xerogel which also explains the lower surface area of 1922 m^2^ g^−1^ and 1878 m^2^ g^−1^ for [FeN_4_]_0.23_[N_4_]_0.77_N_32_C_520_ and [FeN_4_]_0.17_[N_4_]_0.83_N_32_C_520_, respectively.

**Figure 1:**
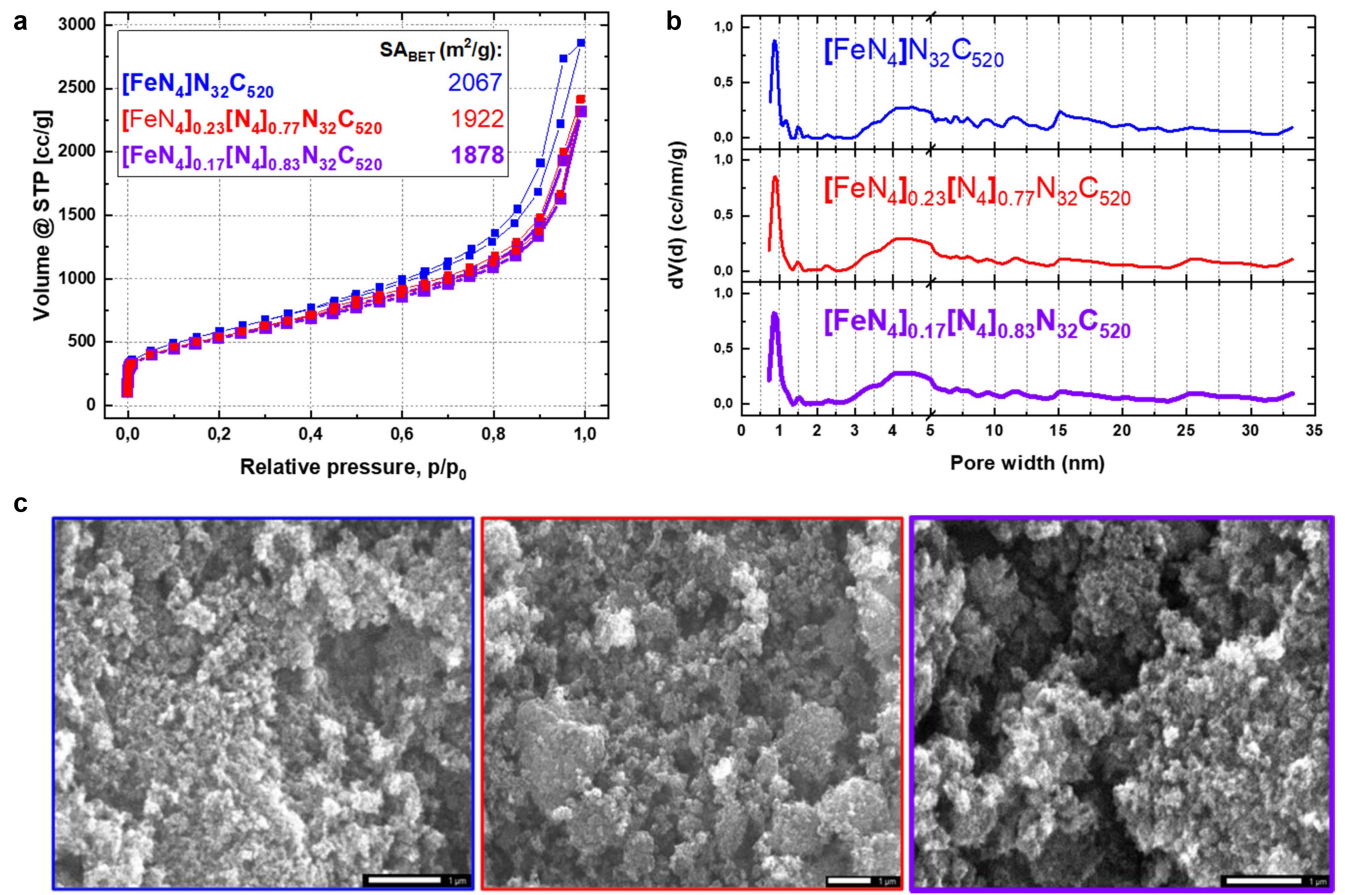
Morphological characterization of the sample in its pristine state and after partial Fe‐extraction. a) Isotherms of the samples obtained from N_2_ sorption measurement and b) the respective pore‐size distribution from the adsorption branch employing the QSDFT model for slit and cylindrical pores. c) SEM images (scale bar: each 1 μm).

Cryo‐Mössbauer spectroscopy measurements of [FeN_4_]N_32_C_520_ at 4.2 K show the presence of two quadrupole doublets with a 3 : 1 intensity ratio. Such doublets are characteristic for atomically dispersed FeN_4_ sites (Figure 2a). The first doublet with an IS of ≈0.25 mm s^−1^ and a QS of ≈1.5–1.7 mm s^−1^ (often referred to as D1)^[11]^ may be explained by low‐spin Fe^2+^ with covalent character or Fe^3+^ and therefore points to O_2_ coordinated Fe^+II^−N_4_ sites. The second doublet (herein for the cryo‐measurements referred to as D2) with an IS of ≈0.74 mm s^−1^ and a QS of ≈3.7–3.8 mm s^−1^ suggests high‐spin Fe^2+^, as in a bare tetrapyrrolic Fe^+II^−N_4_ structure. A magnetically split sextet component is an indication of only 8 % nanoscopic Fe^III^ oxidic clusters. Due to the low relative area of this sextet, these clusters are herein neglected for clarity. Worth nothing is the sharpness of the two doublets, namely a line width of 0.90 mm s^−1^ and 0.55 mm s^−1^ for D1 and D2 respectively. These values are unusual in Fe−N−C catalysts and more common in molecules, likely pointing to a high homogeneity of the FeN_4_ environment.^[17]^ The Mössbauer spectrum of Fe in [FeN_4_]_0.17_[N_4_]_0.83_N_32_C_520_ (Figure 2c), despite its inferior statistical accuracy due to the low iron content of the sample, can be fitted with the same quadrupole doublets as that of [FeN_4_]N_32_C_520_ (Figure 2a), showing that the iron species after extraction are still the same as before. Within the limits of accuracy there is no indication of the presence of a magnetically split pattern due to oxidic clusters. From the ratio of the spectral areas, and taking the slightly different absorber thicknesses into account, one estimates that after extraction the catalyst still contains about 13 % of the iron in the original sample, which tallies well with the results of the ICP‐MS determinations (Table S1). The atomic dispersion of the catalyst was further confirmed by extended X‐ray absorption fine structure (EXAFS) measurements around Fe at the K‐edge (7112 eV). Figure 2b shows the magnitude of the Fourier transformed spectrum of the sample, where one main peak at ≈2 Å is found, typical for Fe−N/O scattering in Fe−N−Cs. A very good agreement with the model based on a 2D tetrapyrrolic Fe−N−C (inset of the figure) is found, allowing for the quantitative extraction of structural parameters. In agreement with expectations based on a general understanding the formation mechanism of Fe−N−Cs, Fe cations in the catalyst are coordinated to four pyrrolic N at 2.04 Å and ≈1 O atoms (from axial OH ligands) at 1.83 Å. The Fe coordination is retained upon partial Fe‐extraction as confirmed by the Fourier transformed EXAFS spectrum of the longest leached [FeN_4_]_0.17_[N_4_]_0.83_N_32_C_520_ (Figure 2d). The lower intensity of the Fe−N/O peak is attributed to the more facile extraction of FeN_4_ sites, which previously contributed with Fe−O bonds due to the additional axial OH‐ligand. The remaining sites are free from oxy coordination, as confirmed by the lack of Fe−O scattering path (Table S4).

**Figure 2:**
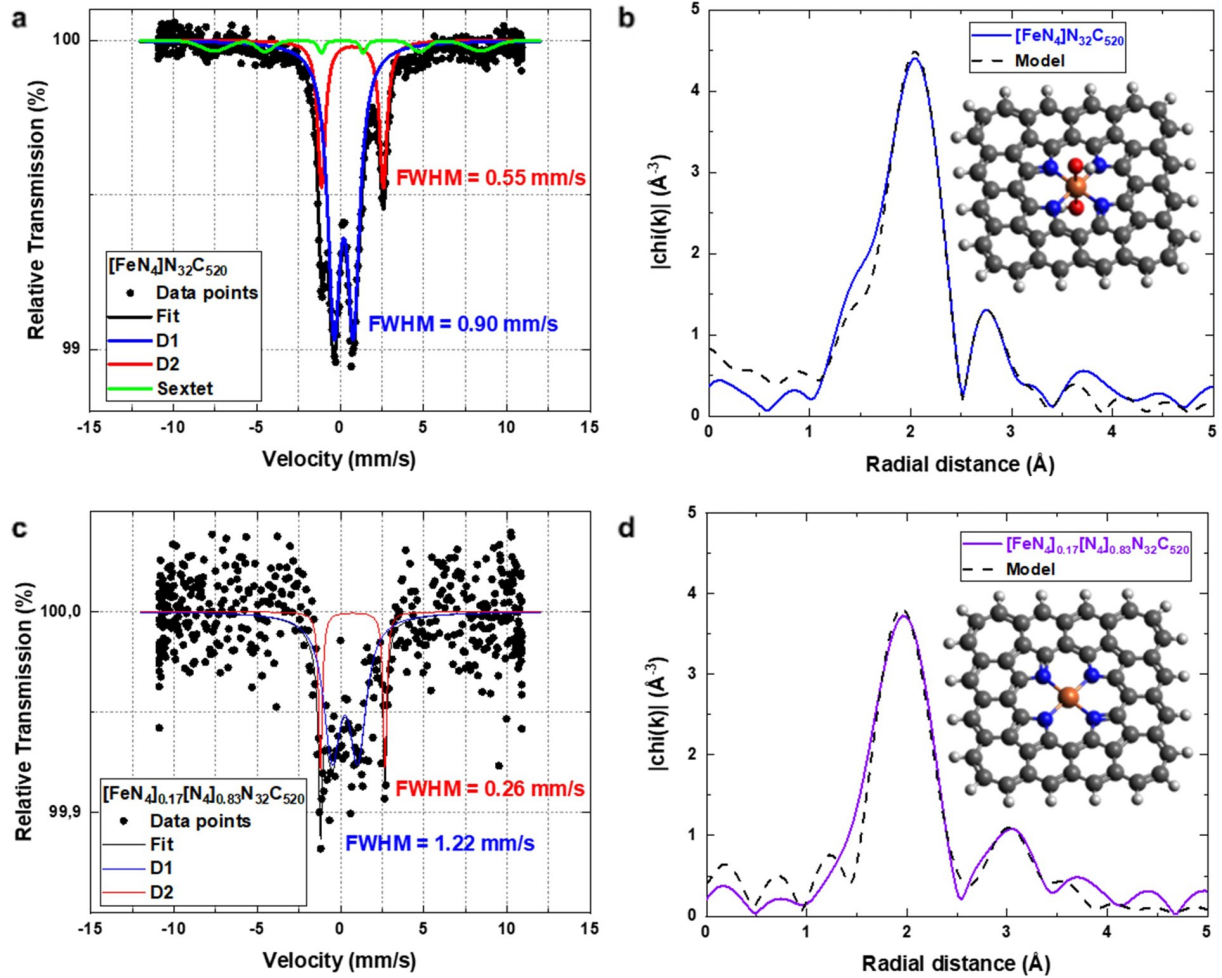
Characterization of the Fe environment of a), b) [FeN_4_]N_32_C_520_ and c), d) [FeN_4_]_0.17_[N_4_]_0.83_N_32_C_520_. a)–c) Mössbauer spectrum measured at 4.2 K; FWHM indicates the line width of the corresponding quadrupole doublet. b), d) Experimental Fourier transform of the Fe K‐edge EXAFS spectra and best fit EXAFS model using the structure displayed in the corresponding inset (orange=Fe; blue=N; red=O; gray=C; white=H).

A rotating disc electrode (RDE) setup was employed to measure the activity and derive the TOF value of the catalysts in O_2_‐saturated 0.1 M HClO_4_ electrolyte. Figure 3a shows the activity of the catalyst in its pristine state and after partial Fe extraction, with the identical catalyst loading of 0.145 mg cm^−2^. Even with this relatively low loading, the catalyst has a good half‐wave potential of 0.70 V_RHE_, which shifts negatively about 140 mV after removing 0.55 wt. %_Fe_ of the initial 0.67 wt. %_Fe_. In terms of mass activity, the catalyst in its pristine state presents a very good value of 2.7±0.3 A g^−1^ at 0.8 V_RHE_. Figure 3b shows the Tafel plots for the pristine and Fe‐extracted samples pointing to equal ORR mechanism before and after extraction. [FeN_4_]N_32_C_520_ has a Tafel slope of ≈60 mV dec^−1^, quantifying the fast kinetics of the catalyst. The comparison of activity after the removal of active metal centres is comparable to the nitrite stripping method, where comparison of activity is carried out before and after active site poisoning. This allows for the TOF calculation of FeN_4_ sites, according to Equation 1.^[10]^ For atomically dispersed Fe−N−Cs, the active site density difference (ΔSD) is calculated simply as the difference in Fe content between the samples (expressed in mol g^−1^), which act as pristine and deactivated states (corresponding to the poisoned state in stripping techniques). This is possible because the extraction naturally targets accessible Fe sites. The gravimetric Fe content should not be used as SD metric due to the potential presence of different Fe phases or buried FeN_4_ sites, although the herein used synthetic method based on active‐site imprinting followed by Zn‐to‐Fe ion exchange promised potential for the exclusive presence of accessible FeN_4_ sites.^[6b]^

(1) $$TOF\;\left[s^{-1}\right]=\frac{\Delta i_{k}\;\left[A\;g^{-1}\right]}{F\;\left[A\;s\;{mol}^{-1}\right]\times \Delta SD\;\left[mol\;g^{-1}\right]}$$

**Figure 3:**
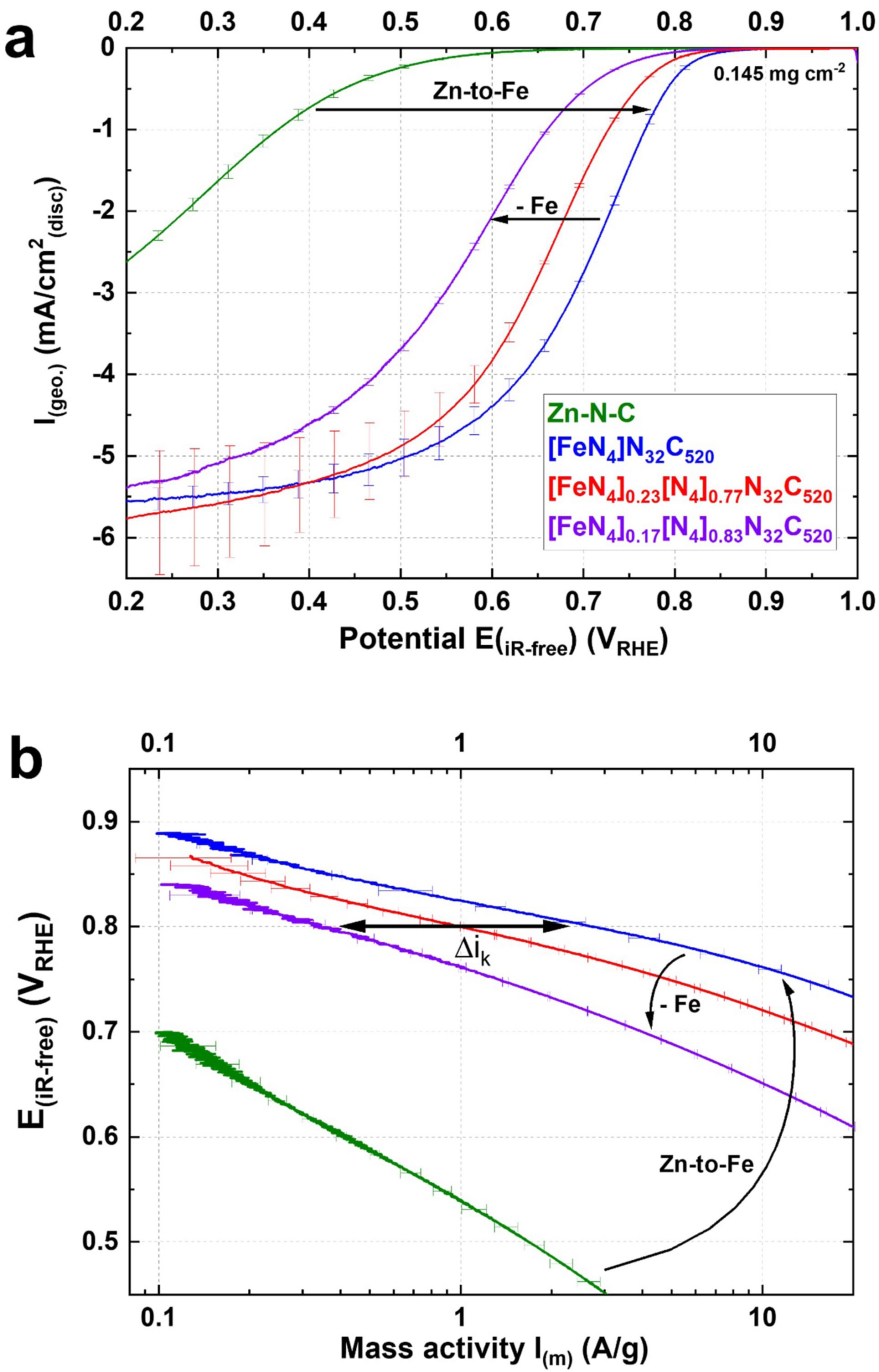
Electrochemical characterization of the sample in its pristine state and after partial Fe‐extraction in comparison with the Zn−N−C material, i.e. the carbon scaffold without any Fe. a) Capacitance‐corrected ORR curves recorded with an RDE setup at room temperature in O_2_‐saturated 0.1 M HClO_4_ at 1600 rpm, 10 mV s^−1^ (anodic scans) and b) respective Tafel plots showing purely the kinetic mass activity corrected for mass‐transport limitation; for Zn−N−C, since no limiting current is achieved, no correction has been applied. The plots clearly show that the carbon scaffold (Zn−N−C) does not contribute to the activity of the catalyst at 0.8 V_RHE_.

Using Equation (1) it is possible to calculate the TOF of the extracted FeN_4_ sites under more realistic PEMFC conditions compared to the nitrite stripping method, i.e. pH 1. For several extracted samples the kinetic current densities can be plotted over the product of molar iron concentration and the Faraday constant. The linear fit reveals the TOF as the slope. At 0.8 V_RHE_, Δ*i*_kin_ and ΔSD have a value of 2.4 A g^−1^ and 9.85×10^−5^ mol g^−1^ when using the longest leached [FeN_4_]_0.17_[N_4_]_0.83_N_32_C_520_. This gives a TOF value of 0.25 s^−1^. The more accurate graphical analysis results in a slope of 0.24 s^−1^ (Figure S3). The TOF value is in line with reported values for Fe−N−C catalyst at this potential, obtained via CO cryo‐sorption and nitrite stripping methods,^[12]^ supporting the validity of the described method. For lower potentials the error is increasing, likely due to contribution of the support material to the current. Assignment of the tetrapyrrolic motif to the active sites by means of EXAFS further allows for a more specific assignment of the average TOF of 0.24 s^−1^ at 0.8 V_RHE_ specifically to tetrapyrrolic FeN_4_ sites. Since this intrinsic activity is below reported specific activity of Fe−N−Cs, the results suggest that more active FeN_*x*_ sites exist.[^8b^, ^10^] Moreover, and not less importantly, based on these results, further considerations about the utilization of the catalyst can be drawn. Keeping in mind that at higher potentials the carbon backbone does not contribute to the ORR activity (Figure 3a), equation 1 can be used to calculate the theoretical kinetic current obtained if all the active sites were available for the ORR (see Supporting Information). Using the gravimetric Fe content as theoretical SD at 100 % utilization in the pristine catalyst and the above‐mentioned TOF of 0.25 s^−1^ obtained via extracting method, a theoretical value of 2.9 A g^−1^ at 0.8 V_RHE_ is calculated. This calculated value is close to the measured value of 2.7±0.3 A g^−1^ obtained at the same potential, showing that the utilization factor corresponds to 93±10 % in RDE measurements, confirming that active sites obtained via transmetalation reaction are highly accessible. Calculation based on the graphical analysis (with a TOF of 0.24 s^−1^) results in a theoretical kinetic current density of 2.8 A g^−1^ and a utilization factor of 96±11 %.

In conclusion, we introduce here a new methodology to derive the specific catalytic activity by means of TOF and utilization of active sites in atomically‐dispersed M−N−C electrocatalysts. In contrast to the established methods like CO cryo‐sorption and nitrite stripping, this method does not rely on probe molecules but solely on the possibility to replace the active‐metal centre with protons. Since no selective binding is involved, the method can be used for any atomically‐dispersed M−N−C catalyst if metal contents are known. The principle of the method is further eliminating questions regarding how many CO molecules bind the active site or how many electrons are transferred in the electrochemical stripping process of NO (i.e., if ammonia or hydroxylamine is formed^[8a]^). Being independent from probe molecules, this method presents several advantages. Compared to CO cryo‐sorption, for example, no harsh pre‐treatments are required and there is no risk of SD overestimation. This is ensured because, due to the conditions of the Fe‐extraction process, all the removed FeN_4_ sites are also accessible to the electrolyte and were hence ORR active.

Compared to the nitrite stripping method, there is no limitation to certain pH values (where the ORR mechanism, and hence the TOF, might be different) or in the concentration of the probe molecule. This allows for the application of this method at operating conditions, i.e. both in acidic and alkaline electrolytes and even at higher temperatures. As long as an extraction of the active metal sites is possible, this method can be extended to all kinds of M−N−Cs and other reactions, holding the potential to push the understanding of this type of materials further.

In the present work the catalyst can be approximated a single‐site catalysts. Hence, the TOF value at 0.8 V_RHE_ is assigned specifically to the ORR activity of tetrapyrrolic FeN_4_ sites (intrinsic activity). Since higher TOF values than 0.24 s^−1^ for Fe−N−Cs were previously reported it may be concluded that other active sites need to be targeted to approach Fe−N−C catalysts for the application in fuel cell electric vehicles.^[5b]^

## Conflict of interest

The authors declare no conflict of interest.

## Supporting information

As a service to our authors and readers, this journal provides supporting information supplied by the authors. Such materials are peer reviewed and may be re‐organized for online delivery, but are not copy‐edited or typeset. Technical support issues arising from supporting information (other than missing files) should be addressed to the authors.

Supporting Information

## Data Availability

The data that support the findings of this study are available in the Supporting Information of this article.
