# Supplementary material for: Evaluation of the Specific Activity of M−N−Cs and the Intrinsic Activity of Tetrapyrrolic FeN4 Sites for the Oxygen Reduction Reaction
Source: Angew Chem Int Ed Engl. 2022 Nov 10;61(50):e202207089. doi: 10.1002/anie.202207089 (PMC10100346; doi:10.1002/anie.202207089)
Supplement: Supplementary file 1 — Supporting Information [file ANIE-61-0-s001.pdf]

Angewandte Chemie
Eine Zeitschrift der Gesellschaft Deutscher Chemiker

Supporting Information

Evaluation of the Specific Activity of M–N–Cs and the Intrinsic Activity of Tetrapyrrolic FeN_4 Sites for the Oxygen Reduction Reaction

*D. Menga, A. Guilherme Buzanich, F. Wagner, T.-P. Fellinger**

Supporting Information

Experimental Section

Preparation of $[FeN_4]N_{32}C_{520}$.

The catalyst was prepared adapting the synthetic procedure reported in previous work.[1] In a typical synthesis, 1 g of 1-ethyl-3-methylimidazolium dicyanamide (Emim-dca) were mixed with 8.26 g of ZnCl2 and 1.74 g of NaCl inside an Ar-filled glovebox. The resulting mixture was placed inside an alumina crucible covered with a quartz lid and heated up in a tube furnace under constant Ar flow. The heating rate was 2.5 K/min and the final temperature of 900 °C was held for 1 hour. Afterwards, the sample was allowed to cool down under Ar flow. The final material was ground and washed with 0.1 M HCl for several hours. After filtering and washing with deionized water until neutral pH was reached, the sample was dried at 80 °C overnight. As previously reported,[2] Zn-to-Fe ion exchange was carried out at low and high temperature. Firstly, the material was degassed at 250 °C under vacuum in a Buchi oven and mixed with a $FeCl_3$/LiCl eutectic mixture inside and Ar-filled flask. After heating the mixture at 170 °C for 5 hours, the sample was washed with deionized water to remove the salt mixture and stirred in 0.1 M HCl for several hours. The powder obtained after further water washing and drying (low temperature Zn-to-Fe exchange) was placed again in an alumina crucible and pushed inside a tube furnace pre-heated at 1000 °C under Ar atmosphere (flash pyrolysis). After 20 minutes the furnace was turned off and opened to achieve a quick cool down of the sample.

Preparation of $[FeN_4]_{0.23}[N_4]_{0.77}N_{32}C_{520}$.

For the partial Fe extraction, $[FeN_4]N_{32}C_{520}$ was placed inside a closed Carius tube and stirred for 16 hours in 2.4 M HCl at 100 °C. After filtering and thoroughly washing with deionized water until neutral pH was achieved, the final sample was obtained.

Preparation of $[FeN_4]_{0.17}[N_4]_{0.83}N_{32}C_{520}$.

For the partial Fe extraction, $[FeN_4]N_{32}C_{520}$ was placed inside a closed Carius tube and stirred for 6 days in 2.4 M HCl at 100 °C. After filtering and thoroughly washing with deionized water until neutral pH was achieved, the final sample was obtained.

Physical characterizations

SEM images were taken with a JEOL JSM-IT200 equipped with a EDX detector. N_2-sorption porosimetry measurements were performed on a Quantachrome Autosorb iQ2 after outgassing the samples at 250 °C under vacuum overnight prior to the measurements. Brunauer–Emmett–Teller (BET) theory was employed to determine the specific surface area using the Micropore BET Assistant supplied by Quantachrome ASiQwin software. Pore size distributions were calculated with the quenched-solid density functional theory (QSDFT) method (slit/cylindrical pores, adsorption branch). Mössbauer measurements at T = 4.2 K were performed on a standard transmission spectrometer using a sinusoidal velocity waveform with both the source of ^{57}Co in rhodium and the absorber in the liquid He bath of a cryostat. In order to refer the measured isomer shifts to α-Fe at ambient temperature, 0.245 mm s^{-1} was added to the measured values. Both X-ray absorption near edge structure (XANES) and extended X-ray absorption fine structure (EXAFS) measurements at the Fe K-edge (7112 eV) were carried out at the BAMline[3] located at BESSY-II (Berlin, Germany), operated by the Helmholtz-Zentrum Berlin für Materialien und Energie. Due to the low concentration of Fe (< 1 wt. %) the measurements were performed in fluorescence mode, with a 4-Element silicon drift detector, using backscattered geometry. For a good S/N ratio, the measurement time was optimized to have at least a total of 500 kcts at the Fe-Kα fluorescence peak. The scans were performed in 10 eV steps until 20 eV before the edge, followed by 0.5 eV steps until 50 eV above the edge, then in 1 eV steps until 200 eV, and from then on in 0.04 Å equidistant k-steps. XANES data evaluation and treatment was performed by using ATHENA program from Demeter package.[4] This includes background removal, energy calibration with Fe metal foil spectrum, and pre- and post-edge normalization. Further information on the local coordination environment was achieved by EXAFS. EXAFS curves were Fourier Transformed between 2-9 Å with a Hanning-type window, to obtain a radial distribution-like information. These were fitted with a model adapted from our previous publication[2] based on a $(OH)_2FeN_4C_{52}H_{20}$ cluster consisting of a Fe atom binding two OH groups in the axial positions and coordinated to four pyrrolic nitrogen atoms embedded in a planar carbon plane. The model was used to calculate the scattering paths by FEFF to be able to quantify the coordination number and bond-length. The goodness of the fit is determined by evaluating the reduced-chi2 test and R-factor. The fiting range was kept between 1-4 Å that includes all the scattering paths displayed in the tables S3 and S4. The degeneracy of the scattering path (which corresponds to the coordination number, N, in the case of single scattering paths) was varied until an amplitude reduction factor (S_0^2) of about 1 was achieved. The best results are displayed the tables S3 and S4. Following amplitude reduction factors were obtained for the two samples: $[FeN_4]N_{32}C_{520}$ ($S_0^2 = 1.0 \pm 0.1$); $[FeN_4]_{0.17}[N_4]_{0.83}N_{32}C_{520}$ ($S_0^2 = 1.0 \pm 0.2$).

Electrochemical measurements

Catalyst inks were prepared by dispersing 5 mg of catalyst in 1.68 mL of N,N-dimethylformamide and 50 µL of 5 wt% Nafion suspension, followed by sonication. To obtained a catalyst loading of 145 µg/cm^2, 10 µL of ink was drop-cast onto a well-polished glassy carbon electrode and dried under an infrared heater for 60 min. The obtained electrodes were measured in a three-electrode glass cell using 0.1 M $HClO_4$ as electrolyte, Au wire as the counter electrode and a freshly calibrated RHE as the reference electrode. The solution resistance was determined by electrochemical impedance spectroscopy. The ORR curves were corrected for capacitive contribution by subtracting from the curves recorded in O_2-saturated electrolyte the ones recorded in Ar-saturated electrolyte. For each curve at least two separate measurements were averaged to give the shown polarization curve, and the standard deviation is illustrated with error bars. Kinetic currents were calculated based on limiting current correction as in reference [5], but may also be evaluated based on Koutecký-Levich analysis.

Calculation of TOF values

For calculation of TOF values from the pristine Fe-N-C and the longest leached Fe-N-C, equation (1) from the manuscript was used. The pristine Fe-N-C contains 8 % of oxidic iron, which is considered in the calculation.

$$TOF\ (s^{-1}) = \frac{\Delta i_k\ (A\ g^{-1})}{F\ (A\ s\ mol^{-1}) \times \Delta SD\ (mol\ g^{-1})} \quad \text{(equation 1′)}$$

$$TOF\ (s^{-1}) = \frac{(2.7 - 0.3)\ A\ g^{-1}}{96480\ A\ s\ mol^{-1} \times \frac{(0.0067\ - 0.0012)\ g}{55.8\ g\ mol^{-1}}} = 0.2524\ s^{-1} = 0.25\ s^{-1}$$

Graphical determination of the TOF value from linear interpolation of the pristine Zn-N-C, the pristine Fe-N-C and two extracted Fe-N-Cs reveals TOF = 0.24143 ± 0.01848 ≈ 0.24 s^{-1} (Figure S3).

Calculation of utilization factor

The utilization factor is defined as the fraction of accessible Fe active sites and the total amount of iron. For convenience both values are usually expressed as molar concentrations. If the catalyst has a homogeneous composition with a known specific activity (mass activity or TOF), it may also be defined as the fraction of the measured activity and the theoretical activity, e.g. the fraction of kinetic current density and the theoretical kinetic current density.

$$utilzation\ factor \equiv \frac{SD\ (mol\ g^{-1})}{molar\ iron\ concentration\ \ (mol\ g^{-1})} = \frac{i_k^{measured}\ (A\ g^{-1})}{i_k^{theoretical}\ (A\ g^{-1})} \quad \text{(equation 2)}$$

Herein the utilization factor can be derived from measured kinetic current density and the product of the Faraday constant, the molar iron concentration and the calculated TOF for tetrapyrrolic Fe-N_4 sites.

$$utilzation\ factor = \frac{\Delta i_k\ (A\ g^{-1})}{TOF \times F\ (A\ s\ mol^{-1}) \times molar\ iron\ concentration\ (mol\ g^{-1})} \quad \text{(equation 3)}$$

The theoretical kinetic current can be calculated as follows:

$$i_k^{theoretical} = TOF \cdot F \cdot SD \quad \text{(equation 4)}$$

$$i_k^{theoretical} = 0.25\ s^{-1} \cdot 96480\ A\ s\ mol^{-1} \cdot \frac{0.0067\ g}{55.8\ g\ mol^{-1}} = 2.896\ A\ g^{-1} \approx 2.9\ A\ g^{-1}$$

From the calculated kinetic current density, finally the utilization factor can be calculated:

$$utilzation\ factor = \frac{i_k^{measured}\ (A\ g^{-1})}{i_k^{theoretical}\ (A\ g^{-1})} = \frac{2.7 \pm 0.3\ A\ g^{-1}}{2.9\ A\ g^{-1}} = \ 93 \pm 10\ \%$$

Additional Figures and Tables

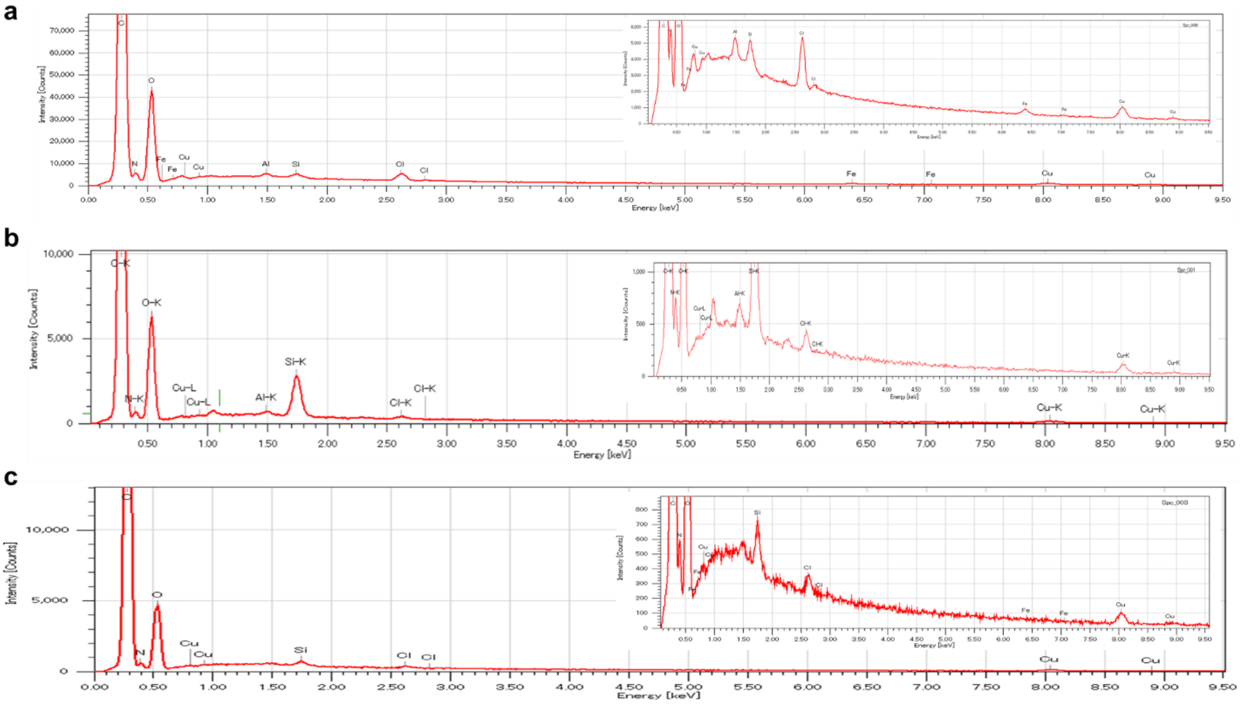

Figure S1. EDX spectra showing the elemental composition of the sample in its pristine state and after partial Fe-extraction. The Cu signal comes from the tape used as a backing for the measurement. a) $[FeN_4]N_{32}C_{520}$, (b) $[FeN_4]_{0.23}[N_4]_{0.77}N_{32}C_{520}$ and c) $[FeN_4]_{0.17}[N_4]_{0.83}N_{32}C_{520}$.

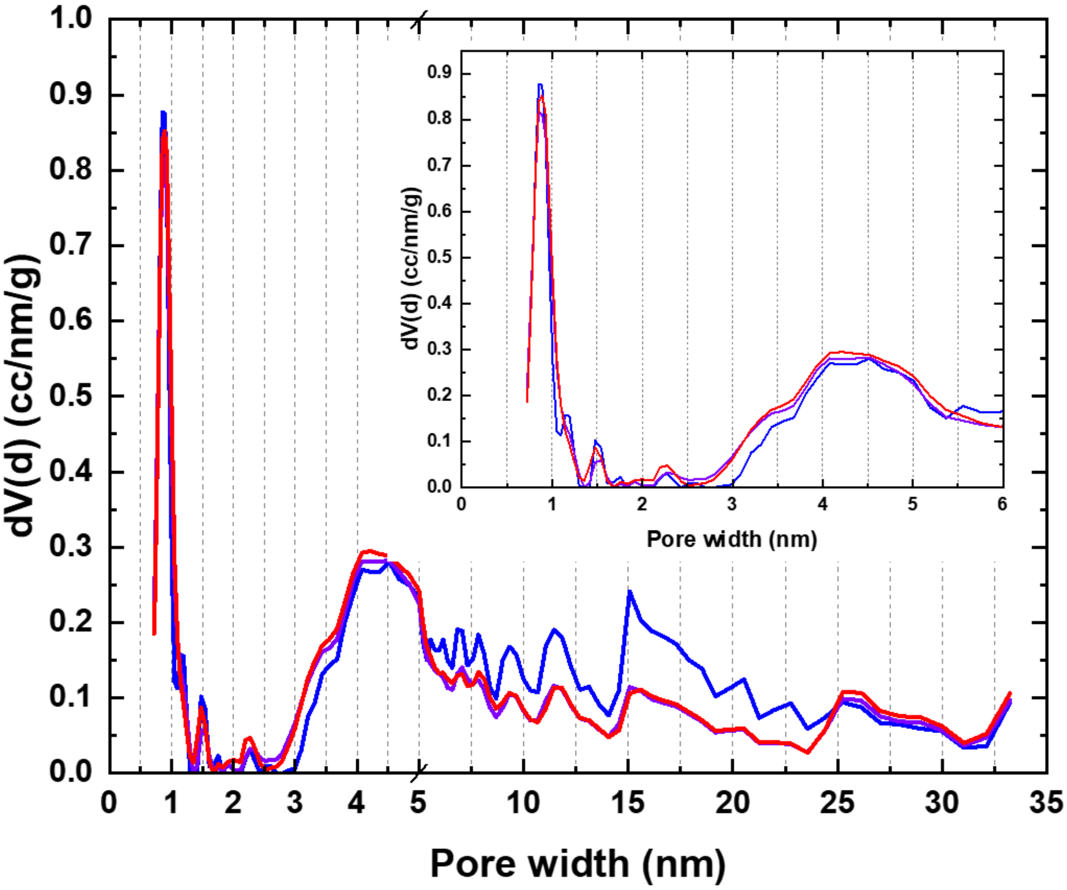

Figure S2. Overlap of the pore-size distribution of the sample in its pristine state and after partial Fe-extraction. The inset shows the region up to 6 nm.

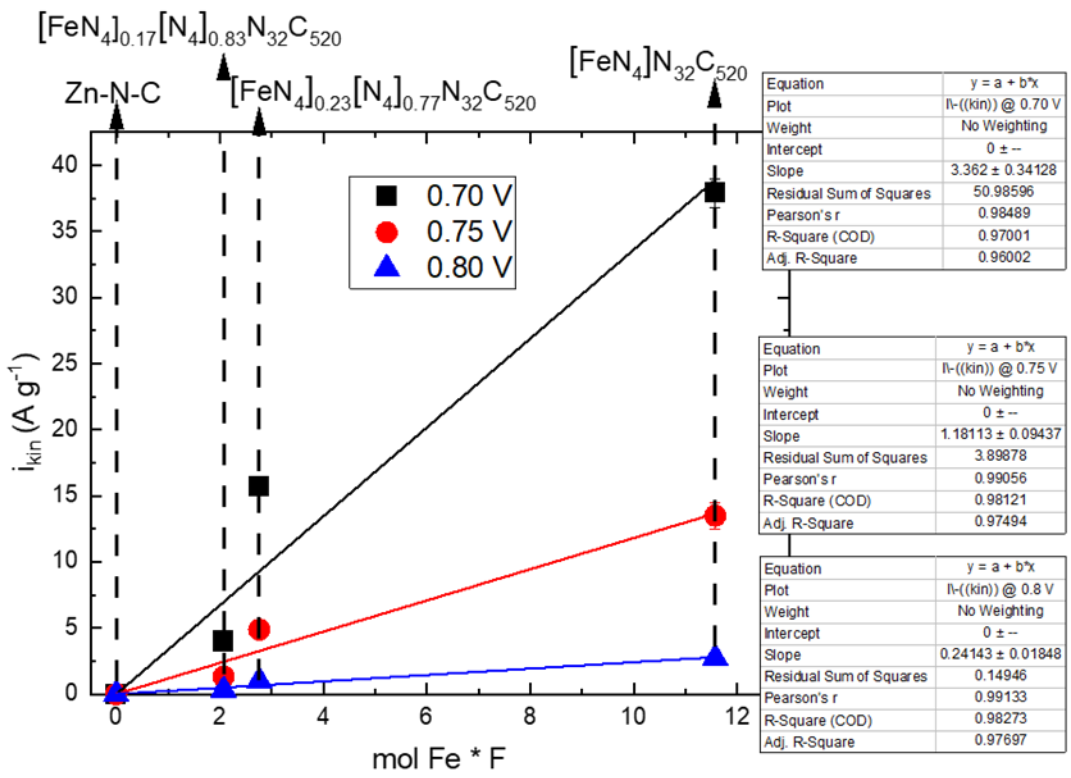

Figure S3. Plot for the TOF calculation. The mass activity at a specific potential is plotted in relation to the molar Fe content. The Zn-N-C sample is also included (0 A g-1 at the three potentials considered and no Fe present). Activity is measured with an RDE setup at room temperature in O_2-saturated 0.1 M $HClO_4$ at 1600 rpm, 10 mV s^{-1} (anodic scans) and the Fe content is measured via ICP-MS analysis. The SD is multiplied by the Faraday constant in order to obtain the TOF value directly from the slope of the line. Slope and R value are reported in the table in the inset.

Table S1. Iron content of the sample in its pristine state and after partial Fe-extraction obtained from ICP-MS analysis.

Sample	Fe (wt.%)
**$[FeN_4]N_{32}C_{520}$**	0.67
**$[FeN_4]_{0.23}[N_4]_{0.77}N_{32}C_{520}$**	0.16
**$[FeN_4]_{0.17}[N_4]_{0.83}N_{32}C_{520}$**	0.12

Table S2. Fitting parameters of the different components obtained from the Mössbauer measurements of $[FeN_4]N_{32}C_{520}$ and $[FeN_4]_{0.17}[N_4]_{0.83}N_{32}C_{520}$ at 4.2 K; isomer shift (IS, mm s^{-1}) with respect to α-iron at 4.2 K, quadrupole splitting (QS, mm s^{-1}), magnetic hyperfine field (H, Tesla), percentage of the spectral area (%) and line width (FWHM, mm s^{-1}).

**Sample**	**D1**<br>**(IS; QS; %; FWHM)**	**D2**<br>**(IS; QS; %; FWHM)**	**Sextet**<br>**(IS; H; %)**
**$[FeN_4]N_{32}C_{520}$**	0.22; 1.17; 69; 0.90	0.74; 3.71; 23; 0.55	0.29; 49.6; 8
**$[FeN_4]_{0.17}[N_4]_{0.83}N_{32}C_{520}$**	0.26; 1.55; 80; 1.22	0.73; 3.88; 20; 0.26	-----------------

Table S3. Structural information obtained from EXAFS by fitting the nearest coordination shells around Fe atoms in $[FeN_4]N_{32}C_{520}$ with model $(OH)_2FeN_4C_{52}H_{20}$: degeneracy of the scattering path (N), interatomic distance from the fit (R) and from the model (Reff), and Debye-Waller factor (σ^2). The goodness-of-fit parameter is indicated by the R-factor.

Scattering path	N	R (Å)	Reff (Å)	σ^2 (Å^2)	R-factor	
Fe-O	1.3	1.83	1.80	0.0036	0.014	Single-scattering
Fe-N	4	2.04	2.01	0.0036		Single-scattering
Fe-C	4	2.99	3.05	0.0116		Single-scattering
Fe-N-C	16	3.20	3.22	0.0181		Triangle scattering

Table S4. Structural information obtained from EXAFS by fitting the nearest coordination shells around Fe atoms in $[FeN_4]_{0.17}[N_4]_{0.83}N_{32}C_{520}$ with model $(OH)_2FeN_4C_{52}H_{20}$: degeneracy of the scattering path (N), interatomic distance from the fit (R) and from the model (Reff), and Debye-Waller factor (σ^2). The goodness-of-fit parameter is indicated by the R-factor.

Scattering path	N	R (Å)	Reff (Å)	σ^2 (Å^2)	R-factor	
Fe-N	4	2.00	2.01	0.0106	0.018	Single-scattering
Fe-C	4	3.02	3.04	0.0067		Single-scattering
Fe-N-C	16	3.22	3.22	0.013		Triangle scattering

Table S5. Surface area (SA) and pore volume (PV) of the sample in its pristine state and after partial Fe-extraction. For the SA, both the value obtained by applying the BET theory (SA_{BET}) and the one from QSDFT calculation with slyt and cylindrical pores (SA_{QSDFT}) are reported. For the PV, both the value from QSDFT (PV_{QSDFT}) and the one measured at $P/P_0 \sim 0.99$ (TPV) are reported. Micro indicates pores ≤ 2 nm and meso pores between 2 nm and 33 nm (upper value of the employed model).

Sample	SA_{BET} ($m^2 g^{-1}$)	TPV ($cc\ g^{-1}$)	SA_{QSDFT} ($m^2 g^{-1}$)			PV_{QSDFT} ($cc\ g^{-1}$)		
			micro	meso	total	micro	meso	total
$[FeN_4]N_{32}C_{520}$	2067	4.42	546	1367	1913	0.238	3.575	3.813
$[FeN_4]_{0.23}[N_4]_{0.77}N_{32}C_{520}$	1922	3.74	557	1184	1741	0.252	2.778	3.030
$[FeN_4]_{0.17}[N_4]_{0.83}N_{32}C_{520}$	1878	3.59	586	1142	1728	0.253	2.667	2.920

Table S6. Elemental composition of the sample in its pristine state. Values for carbon, hydrogen and nitrogen are obtained averaging two independent combustion measurements.

Sample	C (wt.%)	H (wt.%)	N (wt.%)	C/N
$[FeN_4]N_{32}C_{520}$	75.15	1.00	6.08	12.36

References

[1] K. Elumeeva, N. Fechler, T. P. Fellinger, M. Antonietti, *Materials Horizons* **2014**, *1*, 588-594.

[2] D. Menga, J. L. Low, Y.-S. Li, I. Arčon, B. Koyutürk, F. Wagner, F. Ruiz-Zepeda, M. Gaberšček, B. Paulus, T.-P. Fellinger, *Journal of the American Chemical Society* **2021**, *143*, 18010-18019.

[3] H. Riesemeier, K. Ecker, W. Görner, B. R. Müller, M. Radtke, M. Krumrey, *X-Ray Spectrometry* **2005**, *34*, 160-163.

[4] B. Ravel, M. Newville, *J Synchrotron Radiat* **2005**, *12*, 537-541.

[5] U. A. Paulus, T. J. Schmidt, H. A. Gasteiger, R. J. Behm, *J. Electroanal. Chem.*, 2001, *495*(2), 135-145.
